# Supplementary material for: A Socio-Cognitive Review of Healthy Eating Programs in Australian Indigenous Communities
Source: Int J Environ Res Public Health. 2022 Jul 29;19(15):9314. doi: 10.3390/ijerph19159314 (PMC9367833; doi:10.3390/ijerph19159314)
Supplement: Supplementary file 1 [file ijerph-19-09314-s001.zip › ijerph-1783211-supplementary.pdf]

Supplementary file

Table S1. A full summary of studies included in the review

Study	Sample	Intervention	Experimental design and Evidence level	Theory and Measures	Results	Gaps, limitations and/or lessons learned
Good Food Systems Good Food for All Project.<br><br>[48]<br><br>Indigenous authors: not reported<br><br>Funding: National Health and Medical Research Council (Grant #545207)<br><br>Amount: not reported	N=4 aboriginal communities<br><br>Communities ranged in size (250-2000 residents)<br>Majority (> 95%) identifying as Indigenous.	Intervention: Capacity building within the Food System.<br><br>**Behavioural focus:** healthy eating<br>**Intervention strategies:** Building capacity in leadership, traditional food production, food business practices, food practices in community services and food infrastructure.	Longitudinal comparative multi-site case study<br><br>Data collection period: 2009-2013<br><br>III-2	Theory: Nil<br><br>Measures: Store sales (impact on community diet), uptake of action and sustainability, engagement (length of consultations, number of meetings, stakeholder representation, community participation, Indigenous representation)	All communities:<br>Confectionery sales – decreased<br>Water sales – increased<br>Fruit and vegetable intake – no change<br>Soft drinks – no change<br>Community C showed improvements in all food and drink indicators except for relative vegetable sales.<br>Uptake/sustainability: high for actions able to be directly influenced by one party (e.g. a business). Low for activities requiring multi-sectorial involvement.<br><br>Positive engagement in three of four communities	Limitation: Three remote Indigenous communities were managed by non-Indigenous people. This skewed the balance of Indigenous and non-Indigenous attendees.<br><br>Resources needed to be made available across sectors rather than just one narrow food-related body.<br><br>Additional measures are needed to capture food related changes beyond store sales.
SHOP@RIC<br><br>Study Design [49]<br><br>Indigenous authors: not	N=20 stores from remote aboriginal communities in the Northern Territory	Intervention: price discount with or without consumer education strategy.<br><br>**Behavioural focus:** healthy eating<br>**Intervention strategies:**	Randomised Control Trial<br><br>49-week baseline data-collection phase, followed by a 24-week intervention phase (price discount	Theory: Social Cognitive Theory and Social-Ecological Theory<br><br>Measures: Store level (F&V, water, sugary drinks and unhealthy	Price discounts were effective in increasing sales of fruit/veg and bottled water<br>Not effective for diet soft drinks<br>Effect persisted for fruit/veg after removal of discount<br>Consumer education had an additive effect, the greatest	Lessons learned: Young people 19-30 highest consumers. A tailored approach to age might be of benefit.<br>Larger discounts may have stronger results.

Study	Sample	Intervention	Experimental design and Evidence level	Theory and Measures	Results	Gaps, limitations and/or lessons learned
reported<br><br>Funding: Australian National Health and Medical Research Council<br><br>Amount: not reported		1) Price discount (20%) on all fresh and frozen fruit/veg, bottled water and artificially sweetened soft drinks. Discounts were promoted in store.<br>2) Consumer education strategy developed to increase fruit/veg and water intake with monthly themes of health benefits; how much to eat and drink; healthy, quick, and easy meals; supporting family and friends; trying and enjoying new healthy foods; buying more healthy food and making the most of your money	with or without consumer education strategy) and a 24-week post intervention follow-up<br><br>II	foods purchased), impact on total daily dietary energy, contextual measures (population, positioning of foods, price, range, provisions, income, policy, infrastructure), mediation (consumption of F&V, water, sugary drinks and unhealthy foods), self-efficacy, food security, preferences, barriers, the fidelity of the study.	additional benefit was for veg purchases.	Visibility of adding more resources for the program is needed.
Substudy (pre-post of the SHOP@RIC study)<br><br>[50]		Intervention: mediators and moderators of SHOP@RIC study<br><br>**Behavioural focus:** healthy eating<br><br>**Intervention strategies:** Assessed the impacts of store-based mediators and moderators on consequent			Perceived vegetable affordability increased from T1 (19 % to T2 (38 %) and returned to baseline levels at T3. High self-efficacy to eat more fruit and vegetables and to drink less soft drink decreased from T1 to T3. A reduction in soft drink intake of 27% was reported at T3 compared with T1; no changes with time were	

Study	Sample	Intervention	Experimental design and Evidence level	Theory and Measures	Results	Gaps, limitations and/or lessons learned
		diet behaviour. The assessed intake of fruit, vegetable, water and sweetened soft drink.<br>1) Mediators – perceived affordability and self-efficacy<br>2) Moderators – barriers and food security			observed for all other outcome measures<br><br>No significant associations with mediators or moderators for F&V, water and soft drink intake	
Healthy Choice Rewards<br><br>[51]<br><br>Indigenous authors: Project staff, one whom was Torres Strait Islander woman.<br><br>Funding: not reported<br><br>Amount: not reported	N=1 community in far North Queensland (2500km from a major city) (1400 residents)	Intervention: Changes in the food system through incentives and sales<br><br>**Behavioural focus:** fruit and vegetable intake<br><br>**Intervention strategies:** The Healthy Choice Rewards (HCR) program offered community store customers an incentive of a fruit and vegetable voucher to the value of AUD 10 each time a set minimum amount was spent on fruit and vegetables.	Mixed methods approach.<br>Phase one, 15 weeks, trialled a $20 spend of fresh F&V followed immediately by phase two trialled a $15 spend of fresh F&V for 17 weeks.<br><br>III-3	Theory: Nil<br><br>Measures: Acceptability, community perceptions of healthy eating, barriers, feedback through interviews, voucher uptake, implementation, fruit and vegetable sales	Acceptability of the HCR: 61% of respondents were encouraged to consume more fruit and vegetable with the discount.<br>Perceptions of healthy eating were seen as important for young women with children<br>Barriers: vouchers did not decrease the high cost of food.<br>Voucher redemption rates 28.6%<br>Total of 2150 vouchers for the study used<br>Higher redemption in phase two (30%) compared to phase 1 (27%)<br><br>Voucher incentive was not successful in increasing F&V uptake from previous years and was decreased by 7%<br>Implementation: four of the six staff had issues with the	The limitations of this study are that it was conducted in one remote community only and for a short time period, with limited staffing.

Study	Sample	Intervention	Experimental design and Evidence level	Theory and Measures	Results	Gaps, limitations and/or lessons learned
					redemption, limited time to prepare, not enough staff. Non-significant reductions in sales of vegetables and overall food and drink sales were also observed	
[52]<br><br>Indigenous authors: not reported<br><br>Funding This work was supported by the National Health and Medical Research Council (NHMRC) [320860]<br><br>Amount: not reported	N=6 Indigenous communities (n=54 participants) across Northern Tertiary and Western Australia	Intervention: price discount using four different strategies<br><br>**Behavioural focus:** healthy eating<br><br>**Intervention strategies:** Pricing, infrastructure 4 x food and beverage price discount strategies:<br>1. Reduced price on grocery products.<br>2. Reduced price on fresh fruit and vegetable<br>3. Fresh fruit and vegetables at landed cost and<br>4. Diet soft-drink discount: a reduced mark-up on diet soft drinks.	Case control study<br><br>Data collection period: Quantitative data July 2009 – July 2010<br><br>Qualitative data 2011<br><br>III-2	Theory: Nil<br><br>Measures: Impact on sales, implementation fidelity	No discernible impact of the discounts on groceries, fresh fruit and vegetables, and diet soft drinks was detected on store sales/turnover. Non-significant reduction of diet soft drinks; however, there were reductions of similar magnitudes in all drink categories.<br>Change in sales - nil	Limitation: Discounts must be made explicit to customers, and that promotional material is simple, colourful and pictorial. Participants also reported the need for the current and previous prices and the savings to be included.
School Breakfast Programs.	N= 2 schools in rural Western Australia.	Intervention: School education program	Case control study<br><br>Data collection	Theory: Nil	Low uptake from staff to implement changes	Lessons learnt: Better engagement and support for community volunteers would

Study	Sample	Intervention	Experimental design and Evidence level	Theory and Measures	Results	Gaps, limitations and/or lessons learned
[53]<br><br>Indigenous authors: not reported<br><br>Funding: not reported<br><br>Amount: not reported		**Behavioural focus:** breakfast eating<br><br>**Intervention strategies:** An intervention to increase health education, social interaction and learning about nutrition and food origins.	period: February to July 2016<br><br>III-2	Measures: operations within the school, environmental changes within the food system (improving education, eliminating menu items), education, social interaction, experience.	Increase in play and socialisation of food choices before food was served.<br>Procurement of juice makers to increase weekly F&V intake	seem an important strategy, and yet is particularly difficult in the disadvantaged environments in which the schools examined in this study operate.<br>Limited storage of fresh foods in rural and remote areas of Australia has been shown to influence access to healthy foods
Bindjareb Yorgas Health Programme (BYHP)<br><br>[54]<br><br>Indigenous authors: second author Indigenous<br><br>Funding: not reported<br><br>Amount: not reported	N=17 women aged between 18 and 60 years in Western Australia<br><br>Setting: Regional Bindjareb community in the Nyungar nation of Western Australia	Intervention: cooking and nutrition classes<br><br>**Behavioural focus:** healthy eating<br><br>**Intervention strategies:** Recommendations of different food groups to maintain health, development of skills in purchasing healthy food on a budget including reading labels, improve the safe delivery of family foods through safe handling and storage, develop new skills and knowledge in food preparation and cooking and	An ethnographic action research study<br><br>Data collection period: September 2012 – September 2013. School terms over 12 months<br><br>IV	Theory: Guided by the Making Two Worlds Work (MTWW) framework<br><br>Measures: Participants' experiences of the BYHP program	Experiences of overwhelming loss of traditional knowledge.<br><br>Acknowledging collective shame due to confusion about western foods<br><br>Change is too hard, budget constraints, family pressure and convenience of unhealthy food choices<br>Crippled by lack of resources, mistrust and tensions<br><br>Community control empowering the individual through engagement<br><br>Learning for life purpose	Suggested principles for developing cooking and nutrition interventions are considering community needs, understanding the impact of historical factors on health, understanding family and community tensions, and engaging in long-term partnerships to develop community determination.

Study	Sample	Intervention	Experimental design and Evidence level	Theory and Measures	Results	Gaps, limitations and/or lessons learned
		influencing family healthy food choices			Plan for community determination	
FOODcents. Study design<br><br>[55]<br><br>Indigenous authors: not reported<br><br>Funding: Western Australian Department of Health (C05669).<br><br>Amount: not reported	N=875 (total)<br><br>N=706 (non-Indigenous)<br><br>N=169 (Indigenous)	Intervention: Adult nutrition education program.<br><br>**Behavioural focus:** healthy eating<br><br>**Intervention strategies:** single-session or multi-session courses to increase knowledge and motivation to increase consumption of fruit, vegetables and cereals; decrease consumption of foods high in sugar, fat, and salt; and improve healthy food expenditure	Case series (pre-post evaluation)<br><br>2-year timeframe<br><br>III-2	Theory: Nil<br><br>Measures: Knowledge and behaviour changes (confidence, nutrition behaviours)	Confidence - increased<br>Diet-disease knowledge – increased (both groups)<br>Food related knowledge & practices – increased (both groups) in all areas except recommended meat consumption (Indigenous), and recommended dairy consumption (non-Indigenous) and aspects of label interpretation (both groups);<br>Consumption – increased vegetable consumption (both groups); increased fruit consumption (Indigenous only); decreased fast food consumption (both groups)<br><br>Improvements were significantly greater among Indigenous participants for all measures.	Limitations: only included participants with sufficient literacy to complete the survey<br>Identifies a need to examine cost-effectiveness nutrition education programs that cater to Aboriginal populations
Follow-up evaluation of FOODcents<br><br>[56]	N=87 (Indigenous status not known)		Pre-Post-follow-up 2-year timeframe, 4-year follow-up		Improvements maintained for:<br>Confidence	

Study	Sample	Intervention	Experimental design and Evidence level	Theory and Measures	Results	Gaps, limitations and/or lessons learned
Indigenous authors: not reported<br><br>Funding: Western Australian Department of Health (C05669)<br><br>Amount: not reported					Several dietary behaviours Improvements not maintained for:<br>reported intake of fruit or frequency of fast food consumption but maintained for vegetable consumption.	
